# Nostoc commune-derived scytonemin induced mitochondrial cell death in leukemia models

**DOI:** 10.1007/s12032-025-02890-3

**Published:** 2025-07-17

**Authors:** Simona Zilakova, Martina Gavurova, Dominika Sebova, Michal Goga, Martin Backor, Viktoria Medvecova, Dajana Kecsey, Martin Kello

**Affiliations:** 1 Department of Pharmacology, Faculty of Medicine, Pavol Jozef Šafárik University, Trieda SNP 1, 040 11 Košice, Slovakia; 2 Department of Plant Biology, Institute of Biology and Ecology, Faculty of Science, Pavol Jozef Šafárik University, 041 67, Košice, Slovakia; 3 Department of Biochemistry and Biotechnology, Institute of Biotechnology, Faculty of Biotechnology andFood Sciences, Slovak University of Agriculture, Tr. A. Hlinku 2, 949 76 Nitra, Slovakia; 4 Center for Interdisciplinary Biosciences, Technology and Innovation Park, Pavol Jozef Šafárik University in Košice, Jesenná 5, 041 54 Košice, Slovakia

**Keywords:** Scytonemin, Apoptosis, Signal pathways, Tumor, Cancer

## Abstract

Cyanobacteria have long attracted scientific interest through their potential application in the development of new therapeutic approaches, particularly those related to the treatment of cancer. In this study, the antiproliferative effects of *Nostoc commune* extract (NOS) and the cyanobacterial compound scytonemin (SCY) were evaluated against a variety of in vitro cancer models, including cervix, colon, breast, lung, and leukemia cell lines, using resazurin assays. Both of the studied compounds were found to have inhibited metabolic activity in a dose-dependent manner, with IC_50_ values ranging from 60.5 to 462.0 µM for SCY and 157.0 to 740.3 µM for NOS. SCY displayed higher levels of inhibitory activity than NOS against all of the tested cancer models, but was particularly effective against HL-60 and Jurkat leukemia cells, with IC_50_ values recorded as 60.5 µM and 88.2 µM, respectively. However in contrast, the two compounds exhibited significantly lower levels of inhibition against non-cancerous MCF-10A and BJ-5ta cells. Flow cytometry studies of leukemia cells treated with SCY revealed that the compound had effectively inhibited cell proliferation over prolonged periods; HL-60 cells displayed G1 phase arrest which lasted for 48 h, while an accumulated G0/G1 sub-population was detected in Jurkat cells, as indicator of apoptosis. Further analysis of cells treated with SCY observed reduced levels of Rb protein and an increase in p21 expression in both HL-60 and Jurkat cell lines. Apoptotic markers such as phosphatidylserine externalization were observed, and mitochondrial dysfunction characterized by the dissipation of mitochondrial membrane potential was also detected. SCY activated the mitochondrial apoptotic pathway, inducing cytochrome *c* release and subsequent caspase-9, -3, and -7 activation. Finally, PARP cleavage, a typical marker of apoptosis, was identified in both leukemia cell lines following treatment with SCY. The findings suggest that SCY induces apoptosis in leukemia cells through the activation of the mitochondrial pathway, highlighting its potential for development as a future anti-cancer agent.

## Introduction

According to the most recent data available from the GLOBOCAN database, leukemia was the second most common hematological malignancy worldwide in 2022, with only non-Hodgkin lymphoma being diagnosed more frequently. Acute myeloid leukemia is the most prevalent form in adults, whereas acute lymphoblastic leukemia is the most common type among children [1]. Various factors can limit the success of conventional anticancer therapies, such as the heterogeneous nature of tumors, the development of drug resistance, or the occurrence of severe adverse effects. To improve survival rates and improve the quality of life for patients without compromising treatment efficacy unduly, there is a clear need to identify and develop new therapeutic strategies which are less susceptible to the limitations posed by these factors [2].

Recent decades have seen a growing scientific interest in the use of natural products in the development of innovative anticancer agents [3]. More than half of currently available anticancer drugs are derived from plants, marine invertebrates, or microorganisms [4, 5]. One group which has attracted particular interest in pharmaceutical research is cyanobacteria, a large and diverse taxon of gram-negative photoautotrophic prokaryotes. These microorganisms display considerable chemical diversity and stability, and over 1600 molecules have been identified which are produced exclusively by this taxon alone [5].

Cyanobacteria are a significant source of bioactive secondary metabolites which exhibit a wide range of pharmacological activities, and due to their wide global distribution and exceptional biodiversity, they are already widely utilized in biomedical research [6]. More recently, however, research into these microorganisms has placed an increasing focus on the potential of cyanobacterial compounds in the prevention and treatment of cancer. Many compounds identified in cyanobacteria have shown promising anticancer activity, particularly against hematological malignancies such as leukemia, multiple myeloma, and lymphoma. Despite advancements in oncological therapies, however, the effective treatment of hematological cancers remains a major challenge, primarily due to growing resistance to existing anticancer treatments and the relative frequency of relapse [7].

Compounds isolated from algae have demonstrated notable immunomodulatory and anticancer effects in preclinical studies. A prominent example of a clinical agent derived from cyanobacteria is monomethyl auristatin E, a synthetic analogue of the cyanobacterial peptide dolastatin 10, which is used as the cytotoxic component of the antibody–drug conjugate Brentuximab vedotin in the treatment of Hodgkin lymphoma [8].

The cytotoxic effects of cyanobacterial compounds on cancer cells have been extensively studied, with some of these agents exhibiting efficacy at nanomolar concentrations [9]. Among the most significant are cryptophycins, a series of 16-membered macrolides isolated from various species of the *Nostoc* genus which are known for their antimitotic properties. Studies have shown that these compounds deactivate the Bcl-2 protein and induce apoptosis more rapidly and effectively than many anticancer agents in current clinical use [10].

Scytonemin, a photoprotective pigment produced by various cyanobacterial species, was first identified in a study by Zhang et al. as an effective inhibitor of polo-like kinase 1 (Plk1), a key regulator of the cell cycle. Plk1 overexpression is typically associated with aggressive disease progression in numerous malignancies and is often a marker of poor prognosis. The study also found that Scytonemin significantly inhibited the proliferation of multiple myeloma cells and induced cell cycle arrest at the G2/M phase [11].

A reduced form of scytonemin, isolated from the cyanobacterium *Nostoc commune*, had an inhibitory effect on the growth of acute leukemia Jurkat cells by inducing autophagic cell death. Treatment with scytonemin also led to elevated intracellular levels of reactive oxygen species (ROS), resulting in mitochondrial dysfunction in the cell lines [12].

Research has shown that scytonemin functions through a dual mode of action; under certain conditions, it acts as a pro-oxidant by increasing ROS levels, but can also exert an antioxidant effect by scavenging free oxygen radicals, thereby protecting cells from UV-induced oxidative stress [13].

This study aims to investigate the antiproliferative and pro-apoptotic effects of scytonemin on Jurkat T-lymphocyte and HL-60 myeloid leukemia cell lines, with a focus on identifying the mechanisms related to apoptosis induction and mitochondrial dysfunction.

## Material and methods

### Tested compounds

Samples of the cyanobacteria *Nostoc commune* were collected and identified in Antarctica (in January 2020) at James Ross Island close to the J. G. Mendel Polar Station (63°48′02″ S, 57°52′57″ E) by the botanists and lichenologists Dr. Peter Váczi and prof. Martin Backor. The cyanobacteria specimens were deposited in the Herbarium of the P. J. Šafárik Botanical Garden in Košice (KO). Scytonemin (SCY) was prepared using acetone extraction [14] and characterized by HPLC and NMR spectroscopy using the method described in Ručová, 2023 [15]. After isolation and characterization, the extracted SCY was dissolved in dimethyl sulfoxide (DMSO) and diluted directly in a cultured medium (final v/v conc. c ≤ 0.2%). DMSO showed no evidence of cytotoxicity in the cultured cells at the given concentration. The extraction and purification procedures afforded scytonemin in a yield of 15 mg per 1 g of dry *Nostoc commune* biomass, equivalent to a 1.5% (w/w) yield. Due to the low photostability of scytonemin, it was not possible measure ^13^C NMR spectrum and 2D experiments of sufficient quality.

### Cell culture

For the initial screening of scytonemin, the following human cancer cell lines were used: HeLa (CVCL_0030; cervical carcinoma), HCT116 (CVCL_E4IN; colorectal carcinoma), Caco-2 (CVCL_0025; colorectal carcinoma), MCF-7 (CVCL_3397), MDA-MB-231 (CVCL_0062; breast carcinoma), A549 (CVCL_C0DZ; lung adenocarcinoma), HL-60 (CVCL_0002; promyelocytic leukemia), Jurkat (CVCL_0065; T-cell leukemia), MCF-10A (CVCL_0598; non-tumorigenic mammary epithelial cells), and BJ-5ta (CVCL_C8QN; immortalized skin fibroblasts). Adherent cell lines (HeLa, HCT116, Caco-2, MCF-7, MDA-MB-231, A549, MCF-10A, and BJ-5ta) were cultured in appropriate media according to ATCC recommendations. HeLa, MCF-7, MDA-MB-231, and A549 cells were maintained in high-glucose DMEM (Dulbecco’s Modified Eagle Medium; Biosera, Kansas City, MO, USA) supplemented with sodium pyruvate (GE Healthcare, Piscataway, NJ, USA), 10% fetal bovine serum (FBS), and a 1% antibiotic/antimycotic solution (Merck, Darmstadt, Germany). Caco-2 cells were cultured in high-glucose DMEM (GE Healthcare, Piscataway, NJ, USA) supplemented with 10% FBS, penicillin (100 IU/mL), and streptomycin (100 μg/mL) (all obtained from Invitrogen, Carlsbad, CA, USA). HCT116 cells were cultured in a RPMI 1640 medium (Biosera, Kansas City, MO, USA) supplemented with 10% FBS and 1% penicillin/streptomycin solution. MCF-10A cells were cultured in high-glucose DMEM/F12 (Biosera, Kansas City, MO, USA) supplemented with 10% FBS, 1% penicillin/streptomycin, 10 µg/mL insulin, and 0.5 µg/mL hydrocortisone. BJ-5ta cells were maintained in a 4:1 mixture of DMEM and an M199 medium supplemented with 10% FBS and hygromycin B (0.01 mg/mL; Merck, Darmstadt, Germany). Suspension cell lines (Jurkat and HL-60) were cultured in an RPMI 1640 medium supplemented with 10% FBS and 1% penicillin/streptomycin solution. All cells were cultured at 37 °C in a humidified atmosphere containing 5% CO_2_. The viability of the cells exceeded 95% prior to each experiment.

### Resazurin assay

Resazurin breaks down into resorufin in the presence of aerobic respiration in cells, and this principle is used in the Resazurin assay to determine the metabolic activity of cells. Cancer cell lines were seeded at a density of 5 × 10^3^ per well and cultured in 96-well cell plates for 24 h under standard conditions. The cells were then treated and incubated with *Nostoc commune* extract (NOS) and scytonemin (SCY) at concentrations ranging from 5 to 100 µM for 72 h. After this period, cells were incubated with 10 µL of resazurin dye for 1.5 h. Fluorescence was measured using the automated Cytation™ 3 Cell Imaging Multi-Mode Reader (Biotek, Winooski, VT, USA). Excel software (Microsoft 365, version 2302) was used to calculate IC_50_ values by applying a predictive TREND function that used trend lines across a given set of numeric fluorescence values obtained by performing tests across the range of concentrations. Selectivity indexes were calculated as the ratio of half-inhibitory concentrations against non-cancer cells and cancer cells. All experiments were performed in biological triplicates.

### Flow cytometry analyses

Flow cytometry analyses were performed according to the method outlined in the experimental scheme. Leukemia cancer cells (HL-60, Jurkat) were seeded in Petri dishes (25 × 10^4^ cells/dish), cultured for 24 h, and treated with SCY (60.5 µM for HL-60, 88.2 µM for Jurkat) and the DMSO vehicle. The cells were then incubated for 24, 48, and 72 h before being trypsinized and centrifuged. The resulting pellets were resuspended in PBS and separated for multiple analyses. For intracellular staining, the cell population was fixed with 4% paraformaldehyde for 15 min, washed with PBS and permeabilized with 0.5% Tween 20. After 15 min incubation on ice, the cells were stained with the conjugated antibody for 30 min at room temperature in dark conditions. Samples were stained with antibody conjugate/dye (listed below, Table 1) and incubated for 30 min at room temperature in dark conditions. All experiments were performed in biological triplicates. Fluorescent outputs were measured using a FACSCalibur flow cytometer (Becton Dickinson, San Jose, CA, USA). Experimental data was analyzed using Flow Jo 10.0 software (Tree Star, Inc., Ashland, OR, USA, RRID:SCR_008520).

**Table 1:** List of antibody conjugates and dyes used for flow cytometry analyses

Antibody conjugate/dye	Cat. number	Dilution	Company
Cleaved Caspase-9 (Asp315) (D8I9E) Rabbit mAb (PE conjugate)	#31245	1:200	Cell Signalling Technology^®^
p21 Waf1/Cip1 (12D1) Rabbit mAb (PE Conjugate)	#8865	1:200	
Cytochrome *c* Antibody (6H2) FITC Conjugate	#11-6601-82	1:100	Thermo Scientific
Annexin V-Alexa Fluor^®^ 647	A23204	1:100	
CellEvent™ Caspase-3/7 Green Flow Cytometry Assay Kit	C10427	Kit	

### Cell proliferation analysis

The effect of scytonemin on the inhibition of cell proliferation was evaluated using the CellTrace™ Yellow Cell Proliferation Kit (Thermo Scientific, Rockford, IL, USA). Cancer cells were seeded, cultured, and treated with SCY and DMSO according to the method described above. After trypsinization, the cells were centrifuged and resuspended in a 100 µL staining solution, followed by incubation for 20 min at 37 °C in dark conditions. After incubation, the cells were centrifuged, resuspended in culture medium, and seeded in Petri dishes. The cells were then treated with SCY (60.5 µM for HL-60 lines, 88.2 µM for Jurkat lines) and cultured under standard conditions. The cell proliferation rate was measured at 4 different time endpoints (after 0, 24, 48, and 72 h). In each assay, the cells were trypsinized and centrifuged, and the resulting pellets were resuspended in PBS and analyzed using a FACSCalibur flow cytometer. All experiments were performed in biological triplicates. Experimental data was analyzed using Flow Jo 10.0 software and presented in graphical form as histograms and tables showing percentage divided and proliferation and expansion indices. The proliferation index represents the total number of divisions divided by the number of cells involved in the division (with a minimum of 1 division); only responding cells are reflected. The expansion index determines the fold-expansion of the overall culture, in which 1 = input cell count in culture sample.

### Cell cycle analysis

Cell cycle distribution was analyzed using flow cytometry assays on cell lines in which propidium iodide had been incorporated into DNA. Cells were seeded, cultured and treated with SCY and DMSO according to the method described above. At each time endpoint (after 24, 48, and 72 h), cells were trypsinized, centrifuged, resuspended in PBS and fixed with cold 70% ethanol overnight. The cells were then centrifuged and stained with a staining solution composed of 0.5 mg/mL ribonuclease A, a 0.2% final concentration of Triton X-100, and 0.025 mg/mL propidium iodide in 500 µL of PBS (Sigma Aldrich, St. Louis, MO, USA) for 30 min at room temperature in dark conditions. After incubation, flow cytometry analysis of the cell cycle distribution was performed. All experiments were performed in biological triplicates. Experimental data was analyzed using Flow Jo 10.0 software (Tree Star, Inc., Ashland, OR, USA, RRID:SCR_008520) using the Dean-Jett-Fox Model.

### Mitochondrial membrane potential analysis

Flow cytometry analysis was also used to evaluate the disruption of mitochondrial membrane potential. Cells were seeded, cultured and treated with SCY and DMSO according to the method described above. TMRE (Ex/Em 549/574 nm) was added to the cells, followed by incubation for 30 min at room temperature in dark conditions. The FL-2 (585/42) channel was used to collect the fluorescence signal, and the obtained data was analyzed using FlowJo software v.10. All cells (i.e., the untreated controls and those treated with DMSO and SCY) were included in the analysis. All experiments were performed in biological triplicates.

### Western blot

For Western blot analyses, cells were seeded in large Petri dishes (1 × 10^6^ cells/dish) and treated with SCY (60.5 µM for HL-60 lines, 88.2 µM for Jurkat lines) and the DMSO vehicle for 24, 48 and 72 h. A Laemli-Lysis buffer (composed of glycerol, 20% sodium dodecyl sulphate (SDS), 1 M Tris/HCl (pH = 8.6), deionized H_2_O, protease and phosphatase inhibitors; all components obtained from Merck, Darmstadt, Germany) was used to prepare cell lysates. The protein concentrations in each sample were measured using a Pierce^®^ BCA protein assay kit (Thermo Scientific, Rockford, IL, USA) and the automated Cytation™ 3 Cell Imaging Multi-Mode Reader (wavelength 570 nm) (Biotek, Winooski, VT, USA). For the electrophoretic separation procedure, 25 µg of total protein were loaded into each lane and transferred by a dry transfer system (iBlot™ dry blotting system, Thermo Scientific, Rockford, IL, USA) to a PVDF (polyvinylidene difluoride) membrane (Thermo Scientific, Rockford, IL, USA). Total protein levels were visualized using No-Stain™ Protein Labeling Reagent (Thermo Scientific, Rockford, IL, USA) prior to incubation with the antibodies. Membranes were blocked in 5% bovine serum albumin solution (BSA; SERVA, Heidelberg, Germany) for 1 h at room temperature, washed with TBS-Tween and incubated with the primary antibodies (Table 2) overnight at 4 °C. Then, membranes were washed with TBS-Tween (3 × 5 min) and incubated with a secondary antibody (Table 2) for 1 h at room temperature. For the protein detection, iBright™ FL1500 Imaging System (Thermo Scientific, Rockford, IL, USA) was used. Obtained data were normalized to total protein levels [obtained using No-Stain™ Protein Labeling Reagent (Thermo Scientific, Rockford, IL, USA)]. Normalization and quantification of protein expression were performed using iBright Analysis software (version 5.2.2, Thermo Fisher Scientific, Cleveland, OH, USA, RRID:SCR_017632). All experiments were performed in biological triplicates.

**Table 2:** List of WB primary and secondary antibodies

	Cat. number	Mr (kDa)	Dilution	Origin	Company
Primary antibody					
PARP	#9532	116/89	1:1000	Rabbit	Cell Signalling Technology^®^
Phospho Rb	#8516	110	1:1000	Rabbit	
Rb	#9309	110	1:1000	Mouse	
Secondary antibody					
Goat anti-Mouse IgG F(ab′)2 Secondary antibody, HRP	#31436	–	1:10,000–1:200,000	Goat	Thermo Scientific
Goat anti-Rabbit IgG F(ab′)2 Secondary antibody, HRP	#31461	–	1:10,000–1:200,000	Goat	

### Statistical analyses

Experimental data were processed and statistically analyzed using one-way analysis of variance (ANOVA) with Dunnett’s post hoc test through GraphPad Prism software (version 9.0.2., La Jolla, CA, USA; RRID:SCR_002798). Prior to ANOVA, the assumptions of normality and homogeneity of variance in the western blot analysis results were tested and normalized to DMSO. Raw data expressed in percentages were used for the flow cytometry data analysis. Results are presented as a mean ± SD of three independent biological replicates and were considered statistically significant when P values < 0.05 (vs. DMSO treated control).

## Results

### Metabolic activity

The results of the Resazurin assays on a broad spectrum of in vitro cancer models (cervix, colon, breast, lung, leukemia cancer cells) showed that both SCY and NOS had inhibited the metabolic activity of various cancer cells in a dose-dependent manner, with IC_50_ values ranging from 60.5 to 462.0 µM, and 157.0 to 740.3 µM, respectively. As is shown in Table 3, SCY exhibited stronger inhibitory activity than NOS across all in vitro cancer models, with the strongest effect being observed against leukemic HL-60 and Jurkat cells (with IC_50_ values of 60.5 µM and 88.2 µM, respectively). In the case of the non-cancer MCF-10A and BJ-5ta cells, however, significantly lower levels of metabolic inhibition were recorded, with IC_50_ values of 224.1 µM and 197.8 µM for SCY and 2201.4 µM and 1028.4 µM for NOS, respectively. According to the calculated selectivity indexes (Table 4), both of the tested substances showed selectivity towards various cancer cell lines, including leukemia cells, at their half-inhibitory concentrations. No significant inhibition of the metabolic activity was observed in cells treated with the DMSO vehicle at any of the tested concentrations (data not shown). Based on these results, it was decided to conduct further experiments on HL-60 and Jurkat leukemia cell lines treated with SCY.

**Table 3:** Predictive IC_50_ values for NOS and SCY against cancer cell lines

IC_50_	SCY (µM)	NOS (µg/ml)
HeLa	109.2 ± 7.0	740.3 ± 99.0
HCT116	100.0 ± 12.8	559.0 ± 127.2
Caco-2	107.7 ± 1.2	157.0 ± 6.8
MCF-7	283.6 ± 0.7	599.5 ± 120.4
MDA-MB-231	137.7 ± 9.4	528.8 ± 12.9
A549	462.0 ± 38.4	2975.4 ± 554.2
HL-60	60.5 ± 8.5	201.8 ± 45.5
Jurkat	88.2 ± 3.4	679.4 ± 90.3
MCF-10A	224.1 ± 6.8	2201.4 ± 614.6
BJ-5ta	197.8 ± 10.2	1028.4 ± 147.3

Results are presented as a mean ± SD of three independent experiments

**Table 4:** Calculated selectivity indexes (SI) for NOS and SCY related to MCF-10A^a^ and BJ-5ta^b^

	SCY (µM) a/b	NOS (µg/ml) a/b
HeLa	2.1/1.8	3.0/1.4
HCT116	2.2/2.0	4.0/1.8
Caco-2	2.1/1.8	14.0/6.6
MCF-7	0.8/0.7	3.7/1.7
MDA-MB-231	1.6/1.4	4.2/1.9
A549	0.5/0.4	0.7/0.3
HL-60	3.7/3.3	10.9/5.1
Jurkat	2.5/2.2	3.2/1.5

Results are presented as a mean ± SD of three independent experiments

### Antiproliferative activity

**Fig. 1:**
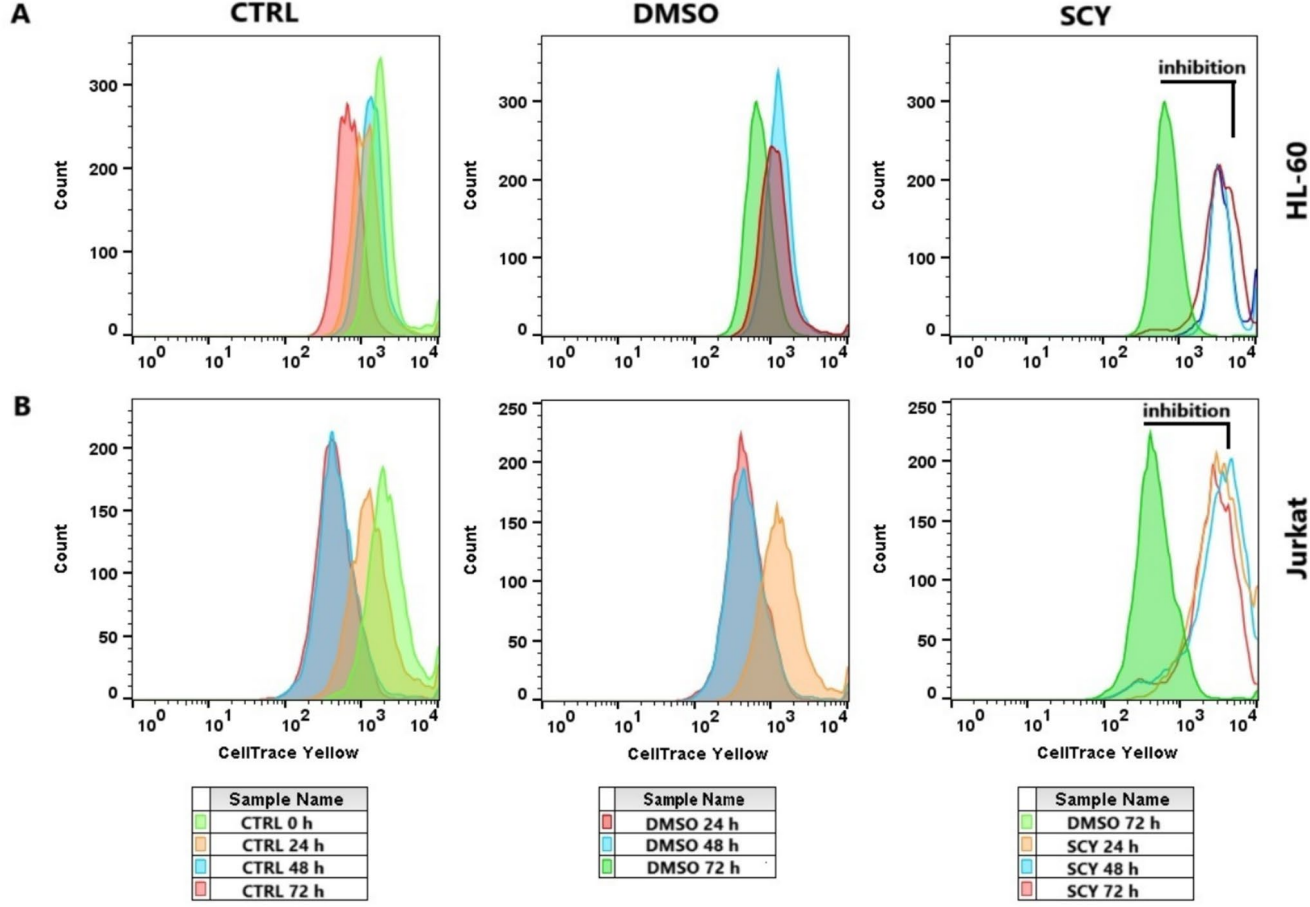
SCY mediated antiproliferative activity. Representative histograms of the division profiles of leukemic HL-60 (**A**) and Jurkat cells (**B**), treated with DMSO vehicle and IC_50_ values of SCY for 24, 48 and 72 h. Results are presented as a mean ± SD of three independent experiments

The effect of SCY on cell proliferation was studied by investigating the incorporation and distribution of the CellTrace™ Yellow dye in multiplying cells. The results of the flow cytometry analyses (Fig. 1, Table 5) show a significant decrease in the percentage of dividing cells in both leukemia cell lines after the application of SCY, a finding which was also reflected in a decrease in the proliferation and expansion indices. The inhibition of proliferation occurred in a time-dependent manner, over the course of 48–72 h. An analysis of cell lines treated with the DMSO vehicle showed normal patterns of cell division as with untreated cell lines.

**Table 5:** Effects of Scytonemin on cell division (%), proliferation and expansion indices against HL-60 and Jurkat cell lines over time

*HL-60*					*JURKAT*				
		Percent divided	Proliferation index	Expansion index			Percent divided	Proliferation index	Expansion index
24	CTRL	26.6 ± 5.9	1.09 ± 0.13	1.35 ± 0.05	24	CTRL	27.2 ± 1.2	1.10 ± 0.1	1.33 ± 0.04
	DMSO	29.7 ± 4.8	1.23 ± 0.17	1.49 ± 0.16		DMSO	26.1 ± 0.9	1.31 ± 0.3	1.41 ± 0.14
	SCY	18.3 ± 0.9	1.02 ± 0.03	1.14 ± 0.06*		SCY	25.9 ± 2.2	1.24 ± 0.04	1.38 ± 0.02
48	CTRL	32.2 ± 2.9	1.00 ± 0.00	1.28 ± 0.09	48	CTRL	41.9 ± 0.7	1.72 ± 0.28	2.10 ± 0.19
	DMSO	36.3 ± 2.0	1.12 ± 0.12	1.35 ± 0.01		DMSO	36.1 ± 2.7	1.23 ± 0.23	1.55 ± 0.22
	SCY	11.8 ± 2.0*	1.00 ± 0.00	1.12 ± 0.02*		SCY	24.4 ± 1.1*	1.00 ± 0.00	1.25 ± 0.02*
72	CTRL	51.9 ± 0.3	1.88 ± 0.13	2.52 ± 0.03	72	CTRL	41.5 ± 1.5	1.36 ± 0.36	1.74 ± 0.31
	DMSO	47.5 ± 2.9	1.66 ± 0.35	1.81 ± 0.42		DMSO	38.2 ± 2.6	1.27 ± 0.17	1.50 ± 0.09
	SCY	6.7 ± 3.2*	1.00 ± 0.00*	1.10 ± 0.02*		SCY	14.1 ± 6.6*	1.00 ± 0.00*	1.20 ± 0.02*

Results are presented as a mean ± SD of three independent experiments (*p < 0.05 compared to DMSO)

### Cell cycle arrest induction

**Fig. 2:**
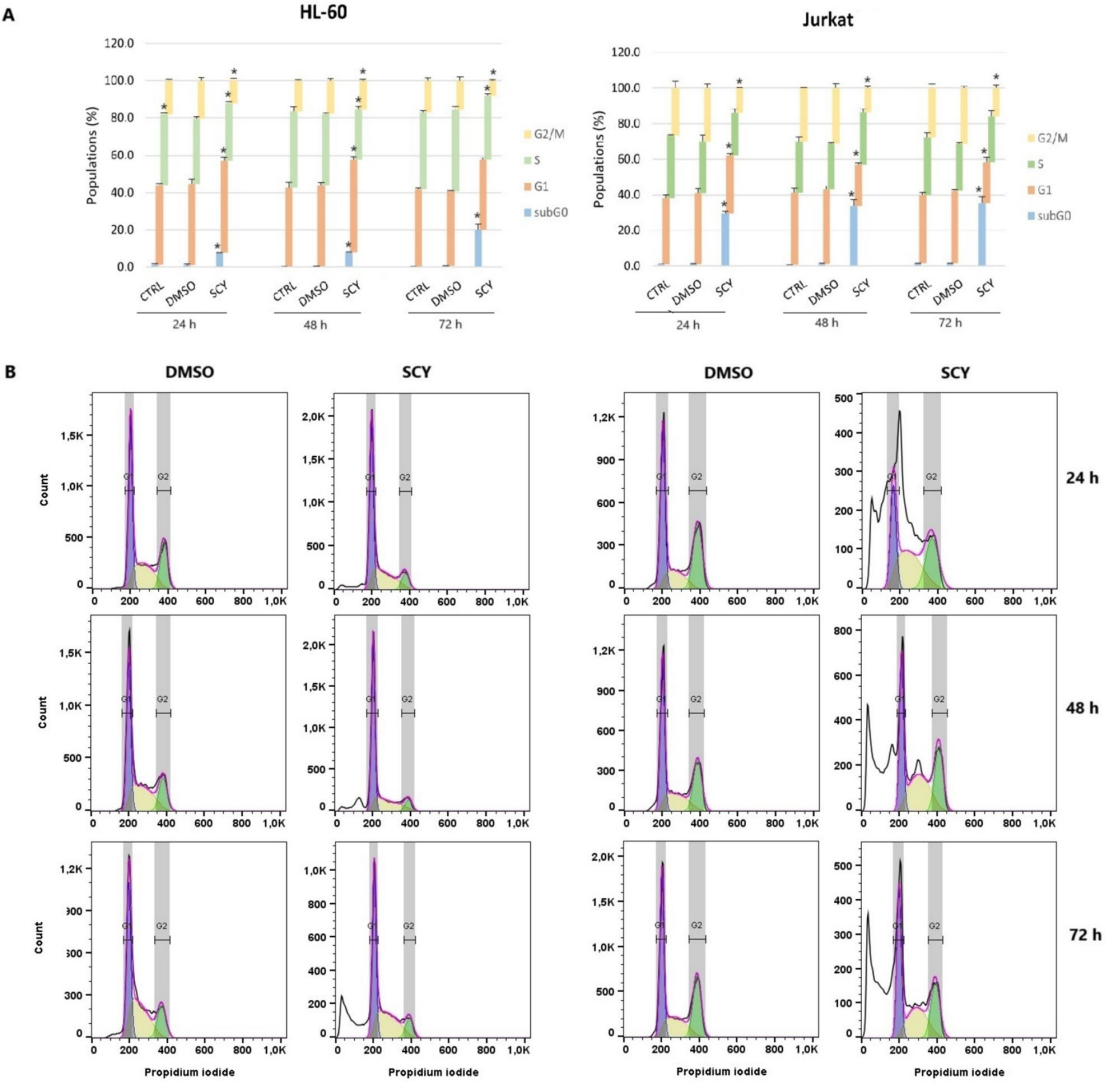
**A** Cell cycle distribution of HL-60 and Jurkat cells treated with IC_50_ of SCY for 24, 48, and 72 h. **B** Representative diagrams of cell cycle distribution of leukemia cells treated with IC_50_ of SCY for 24, 48, and 72 h. Results are presented as a mean ± SD of three independent experiments

To determine the molecular mechanism of the SCY-mediated suppression of cell growth, cell cycle progression in HL-60 and Jurkat cells treated with SCY was investigated over the course of 24, 48, and 72 h (Fig. 2A, B). Flow cytometry analyses of HL-60 cell lines stained with propidium iodide show clear evidence of G_1_ cell cycle arrest; the results also demonstrate that the arrest was sustained at the same level for between 24 and 48 h without any significant increases or decreases. Cell cycle arrest ceased after 72 h of treatment, and this was accompanied by a concomitant increase in the percentage of cells in the SubG_0_/G_1_ phase, a stage of DNA fragmentation which is typically associated with apoptotic cell death. In contrast, however, no cell cycle arrest was detected in the Jurkat cell lines after treatment with SCY, but a significant population of Jurkat cells in the SubG_0_/G_1_ phase was observed after 24, 48, and 72 h of treatment.

### Effect of scytonemin on Rb (cell cycle-related) protein expression

**Fig. 3:**
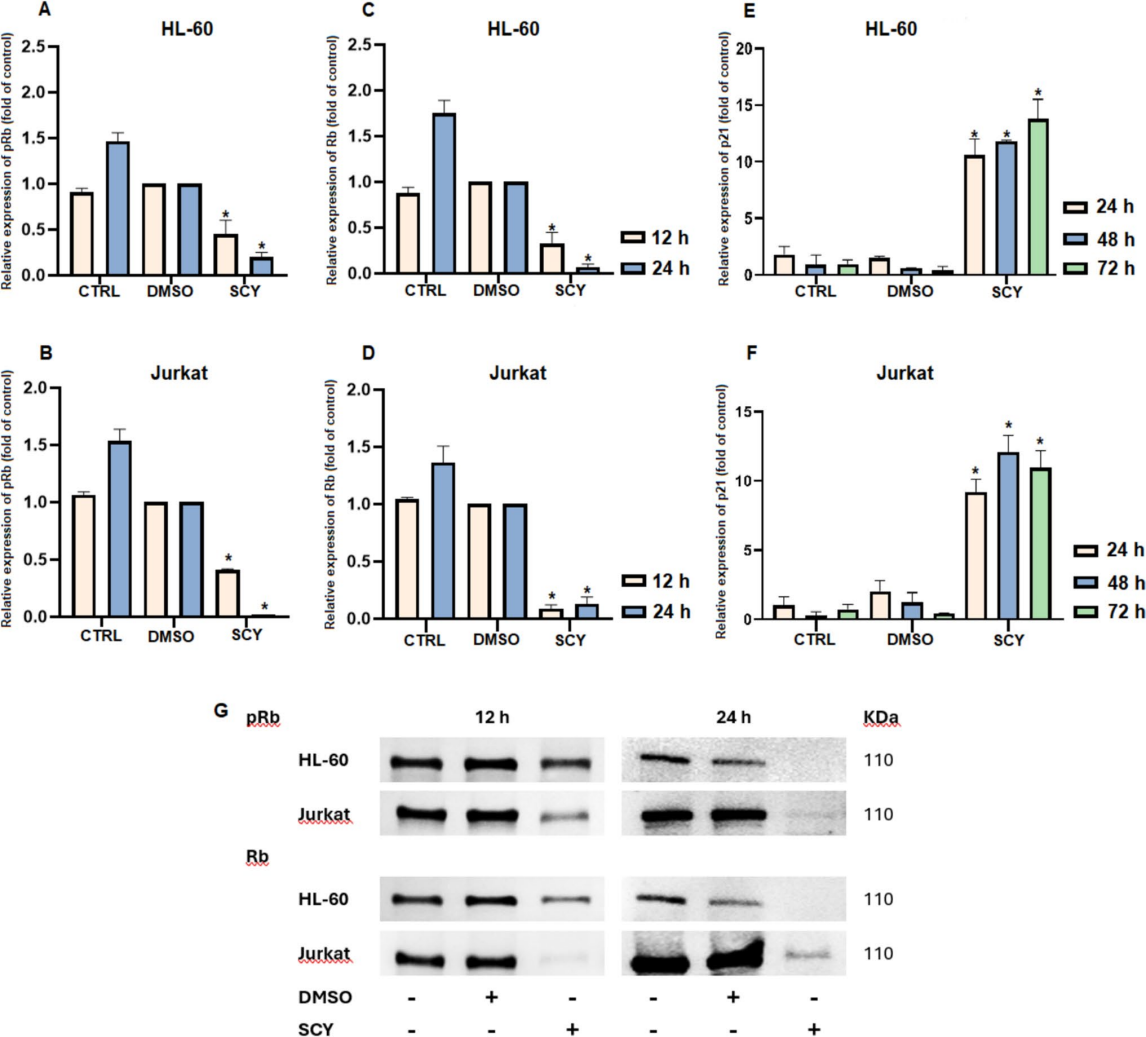
Modulation of pRb, total Rb and p21 protein expression in SCY-treated leukemia cells. Densitometry analysis of relative expression of pRb (**A**, **B**) and total Rb (**C**, **D**) in HL-60 and Jurkat cells. Flow cytometry analysis of relative expression of p21 in HL-60 cells (**E**) and Jurkat cells (**F**). Western blot analysis of pRb and Rb expression (**G**). Results are presented as a mean ± SD of three independent experiments (*p < 0.05 compared to DMSO control, based on ordinary one-way ANOVA with Dunnett’s post hoc test)

Rb protein is a key regulatory factor in the cell cycle and plays a particularly important role in the transition from the G1 phase to the S phase. Rb proteins are released from their complexes with E2F through phosphorylation, and this modification process activates E2F and triggers the transcription of genes required for DNA replication. The results of our analyses revealed reduced levels of both phosphorylated (pRb) (Fig. 3A, B, G) and total (Fig. 3C, D, G) forms of Rb protein in both HL-60 and Jurkat leukemia cell lines exposed to SCY, with changes occurring in a time-dependent manner. In the case of the Jurkat cells, pRb levels had decreased by 58.7% ± 0.6% 12 h after treatment with SCY and by 98% ± 0.0% after 24 h, while total Rb levels dropped by 91% ± 3% and 87% ± 6%, respectively. In the case of the HL-60 cells, pRb levels had decreased by 55% ± 15% 12 h after treatment with SCY and by 80% ± 5% after 24 h, while total Rb levels had decreased by 67.7% ± 12.5% and 93% ± 3%, respectively. Flow cytometry analyses also showed increased levels of p21 expression in both tested cell lines after SCY treatment in a time dependent manner (Fig. 3E, F). A significant reduction in phosphorylated Rb was detected as early as 12 h after treatment, as is shown in Fig. 3A–D; this finding is confirmed by the Western blotting results (Fig. 3G), while a marked induction of p21 expression only became evident after 24 h (Fig. 3E, F).

### Phosphatidylserine externalisation

**Fig. 4:**
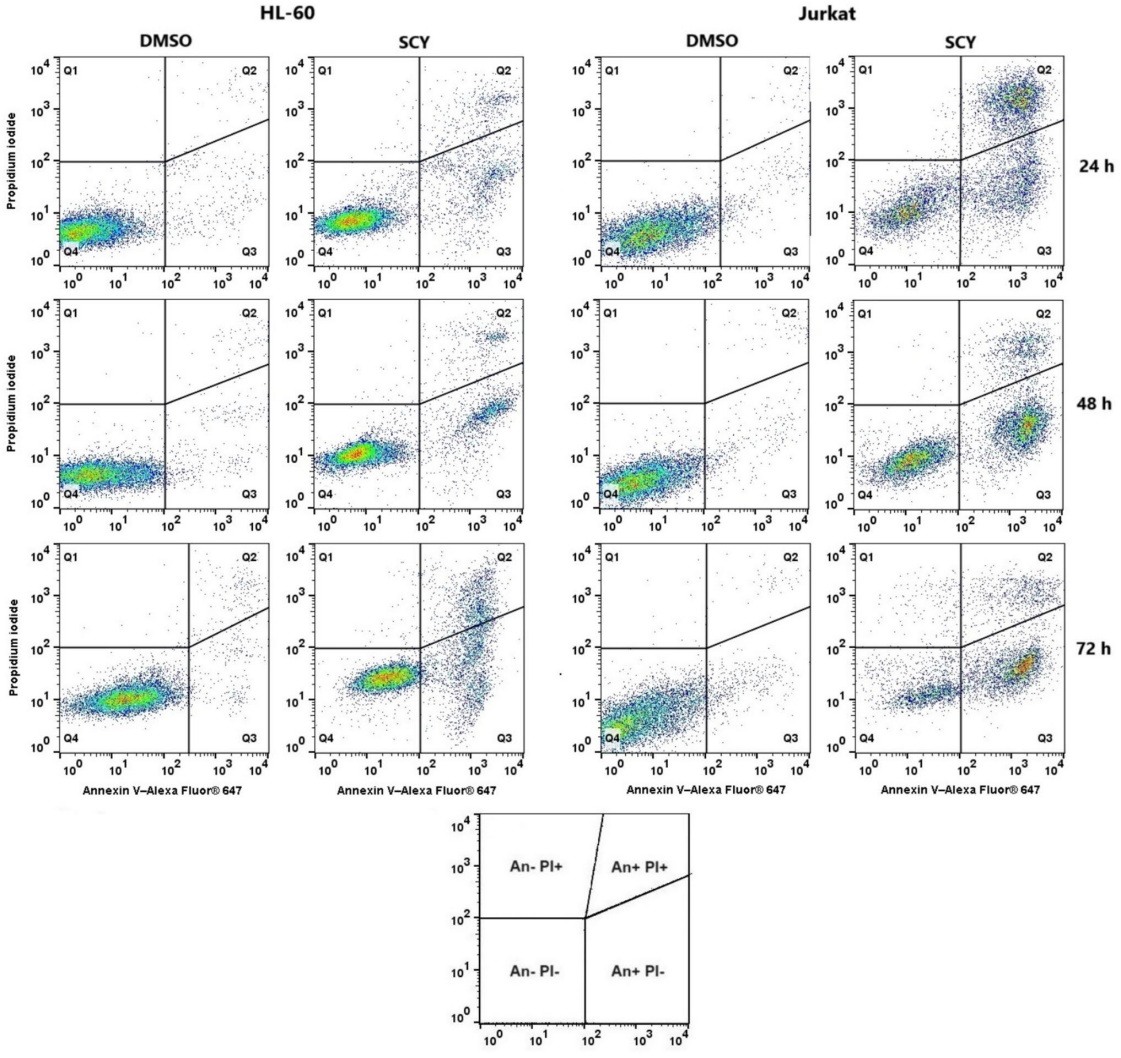
Representative dot plots illustrating apoptotic death in HL-60 and Jurkat cells incubated with IC_50_ of SCY for 72 h

The Annexin V/propidium iodide double staining technique can be used to determine the externalization of phosphatidylserine (PS), an very early marker of the initiation of apoptotic cell death. In our assay, the approach was applied to HL-60 and Jurkat cell lines treated with SCY to identify living cells (An-PI), cells in early (An+PI−) or late (An+PI+) stages of apoptosis, or dead cells (An−PI+) within the cell population. The results presented in Table 6 and Fig. 4 suggest that SCY had treatment resulted in a time-dependent increase in the percentage of cells in early and late apoptotic stages, with a concomitant decrease in a living cell population in both leukemia cell lines. In the case of HL-60 cells, a total apoptosis level 22.1 ± 2.2% was reached at 24 h and 23.4 ± 3.8% at 48 h, with a peak at 30.8 ± 3.3% after 72 h. In the case of the Jurkat cells, a markedly higher level of apoptotic response was detected, with 65.6 ± 2.6% at 24 h, 70.3 ± 9.2% at 48 h, and 67.4 ± 9.2% at 72 h.

**Table 6:** Apoptosis analysis of HL-60 and Jurkat cell lines treated with IC_50_ of SCY for 24, 48 and 72 h

		HL-60			Jurkat		
		CTRL	DMSO	SCY	CTRL	DMSO	SCY
24 h	An-PI-	93.5 ± 0.4	95.0 ± 0.5	77.7 ± 2.1*	95.7 ± 0.4	95.6 ± 0.2	33.2 ± 3.0*
	An + PI-	5.3 ± 0.5*	3.9 ± 0.3	14.5 ± 0.2*	3.5 ± 0.4	3.6 ± 0.1	30.1 ± 1.9*
	An + PI +	1.2 ± 0.2	1.0 ± 0.3	7.6 ± 2.0*	0.7 ± 0.1	0.8 ± 0.1	35.5 ± 0.7*
	An-PI +	0.1 ± 0.1	0.1 ± 0.1	0.3 ± 0.1*	0.1 ± 0.1	0.1 ± 0.1	1.2 ± 0.5*
	SUM Apo	6.5 ± 0.7	4.9 ± 0.6	22.1 ± 2.2*	4.2 ± 0.5	4.4 ± 0.2	65.6 ± 2.6*
48 h	An-PI-	94.7 ± 0.8	94.9 ± 0.2	76.4 ± 2.0*	96.2 ± 0.5	94.2 ± 1.1	29.0 ± 8.8*
	An + PI-	4.4 ± 0.7	4.2 ± 0.4	19.5 ± 2.9*	3.0 ± 0.4	4.8 ± 1.0	58.6 ± 7.6*
	An + PI +	0.9 ± 0.1	0.8 ± 0.1	3.9 ± 0.9*	0.7 ± 0.4	0.9 ± 0.1	11.7 ± 1.6*
	An-PI +	0.0 ± 0.1	0.1 ± 0.1	0.2 ± 0.1*	0.1 ± 0.1	0.1 ± 0.1	0.8 ± 0.4*
	SUM Apo	5.3 ± 0.8	5 ± 0.5	23.4 ± 3.8*	3.7 ± 0.8	5.7 ± 1.1	70.3 ± 9.2*
72 h	An-PI-	95.8 ± 0.9	95.3 ± 0.1	68.5 ± 3.2*	95.7 ± 1.1	95.1 ± 0.6	30.4 ± 9.2*
	An + PI-	2.8 ± 0.1	3.2 ± 0.6	20.8 ± 1.4*	3.9 ± 0.9	4.5 ± 0.4	58.9 ± 9.1*
	An + PI +	1.1 ± 0.7	1.3 ± 0.4	10.0 ± 1.9*	0.3 ± 0.1	0.4 ± 0.2	8.5 ± 0.1*
	An-PI +	0.4 ± 0.3	0.1 ± 0.1	0.8 ± 0.2*	0.1 ± 0.1	0.2 ± 0.1	2.3 ± 0.2*
	SUM Apo	3.9 ± 0.8	4.5 ± 1.0	30.8 ± 3.3*	4.2 ± 1.0	4.9 ± 0.6	67.4 ± 9.2*

The results are expressed as a mean ± SD of three independent experiments (*p < 0.05 compared to DMSO). An-PI- = Live, An+PI− = Early Apo, An+PI+ = Late Apo, An-PI+ = Death

### Mitochondrial membrane potential changes

**Fig. 5:**
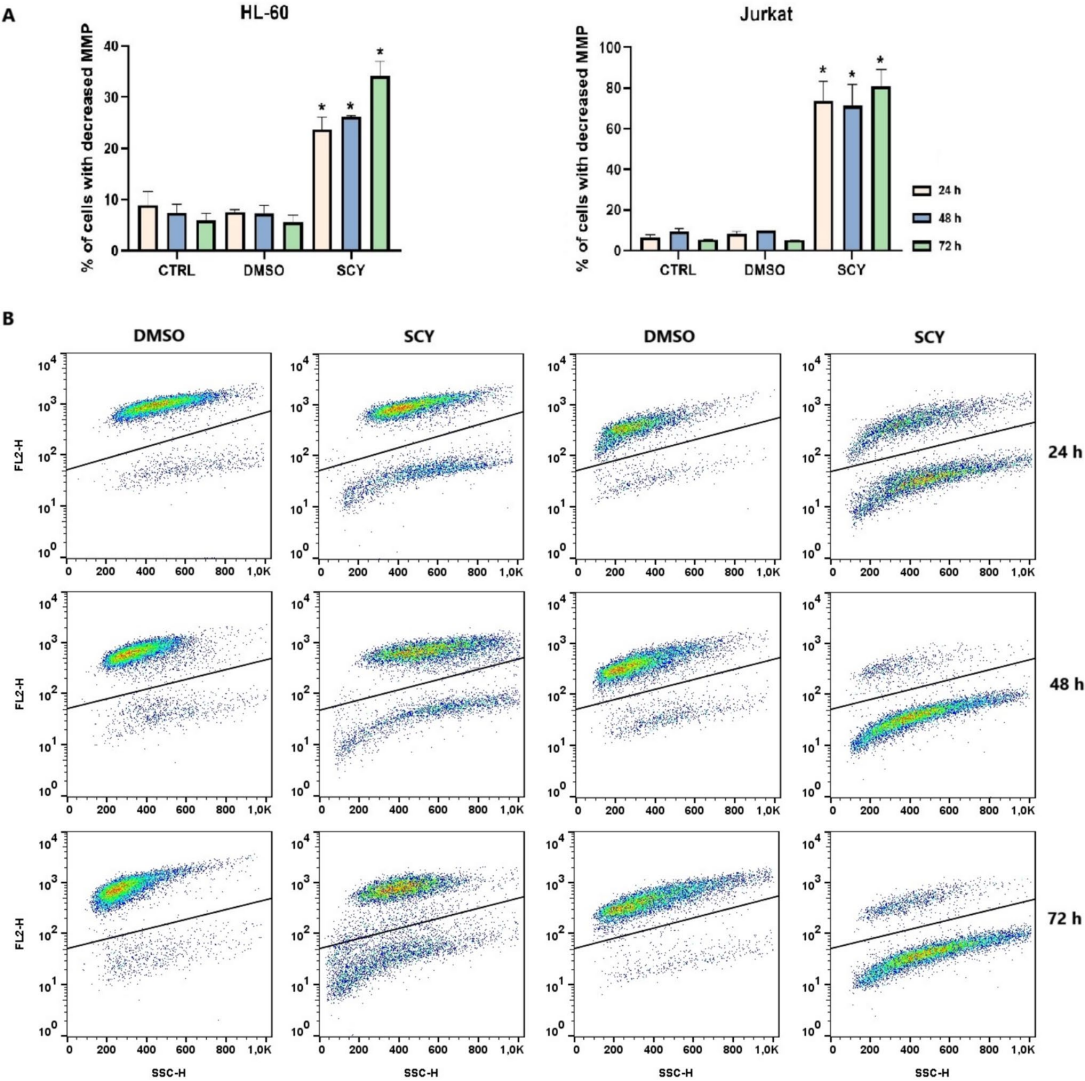
**A** Relative percentage of cells with decreased MMP after 24, 48 and 72 h of treatment with SCY and **B** Representative dot plots (**A**) illustrating disruption of MMP in SCY-treated HL-60 and Jurkat cells after 72 h. Results are presented as a mean ± SD of three independent experiments. (*p < 0.05 compared to DMSO)

TMRE fluorescent probes can be used to assess changes in mitochondrial membrane potential (MMP) as they accumulate in actively functioning mitochondria. The results of our assay are shown in Fig. 5. The findings demonstrate that HL-60 and Jurkat leukemia cell lines treated with SCY exhibited a time-dependent increase in the percentage of cells with dissipated MMP, indicating that mitochondrial dysfunction had been induced. The treatment with SCY was found to be more efficient in the Jurkat cell line, with a higher percentage of cells with decreased MMP being identified in comparison to HL-60 cells.

### Mitochondrial apoptotic pathway activation

The initial cell cycle analysis had recorded a time-dependent accumulation of cancer cells in the Sub-G0/G1 subpopulation as a result of SCY-mediated DNA fragmentation, and, therefore, further analyses were performed to gain a fuller understanding of the molecular mechanisms of SCY-mediated programmed cell death, more specifically of mitochondrial apoptotic pathway activation.

**Fig. 6:**
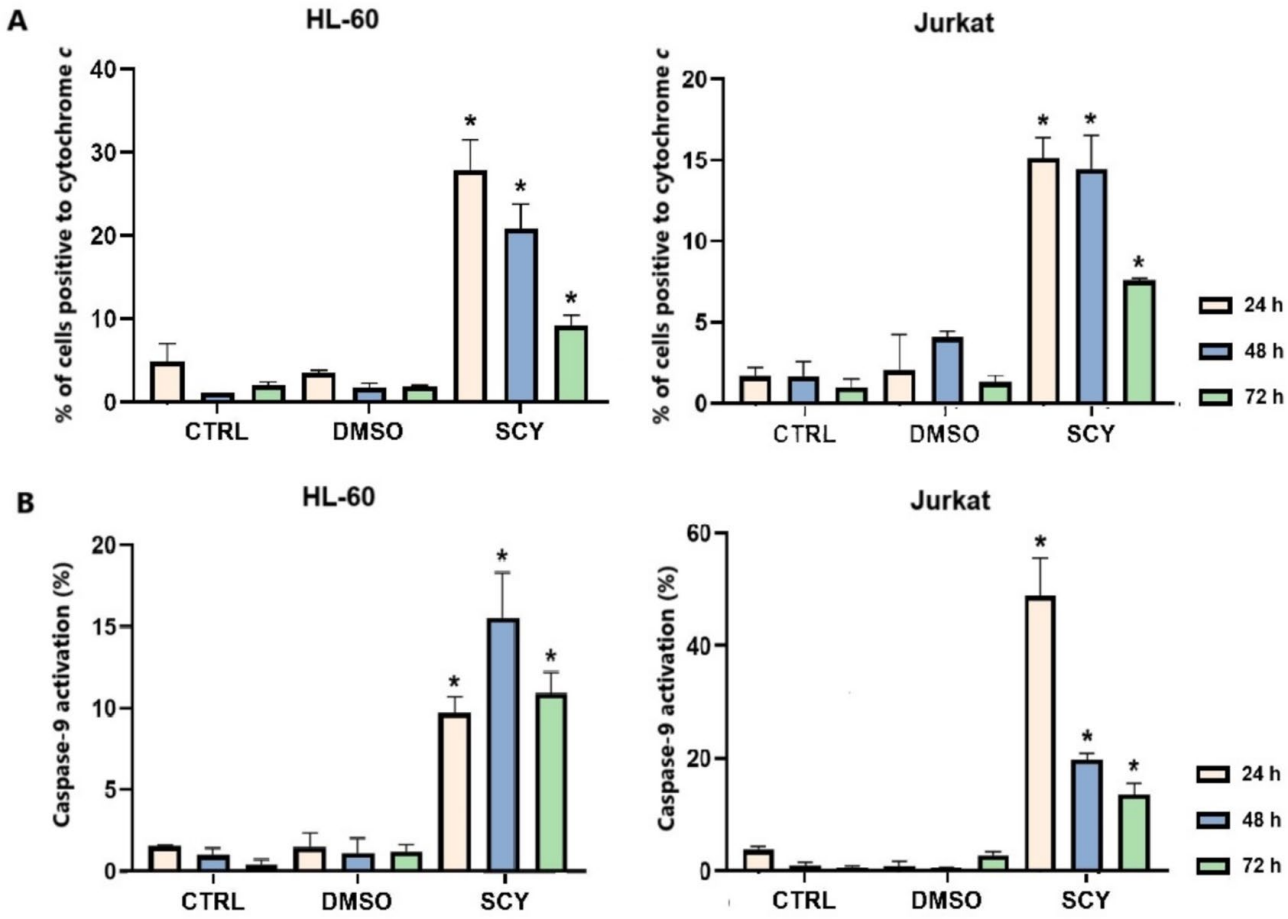
Cytochrome *c* release (**A**) and caspase-9 activation (**B**) in SCY-treated HL-60 and Jurkat cell lines. Cells were treated with IC_50_ of SCY for 24, 48, and 72 h. Results are presented as a mean ± SD of three independent experiments (*p < 0.05 compared to DMSO control, based on ordinary one-way ANOVA with Dunnett’s post hoc test)

Caspase-9 plays a crucial role in triggering apoptosis, and this enzyme is activated when cytochrome *c* is released from the mitochondria and binds with APAF-1 (apoptosome). Our studies of HC-60 and Jurkat cell lines treated with SCY found that cytochrome *c* had been released from an intermembrane space into the cytosol, with maximums for both cell lines being observed after 24 h of treatment (Fig. 6A). Similarly, SCY was also found to have induced caspase-9 cleavage in a time dependent manner after 24 h in both cancer cell lines (Fig. 6B), a process which in turn activated the executioner caspases-3 and -7 (Table 7). Caspase-3/7 was also identified in early and late apoptotic populations of both cancer cell lines in tandem with a time dependent increase in cell populations with lost permeability, a critical state immediately prior to cell death.

**Table 7:** Relative percentage of cells positive to caspase-3/7 and/or SYTOX™ ADvanced™

		HL-60			Jurkat		
		CTRL	DMSO	SCY	CTRL	DMSO	SCY
24 h	Live	95.9 ± 0.3	96.2 ± 0.7	77.2 ± 3.3*	95.9 ± 0.9	96.0 ± 0.8	33.4 ± 2.7*
	Early Apo	1.0 ± 0.2	0.7 ± 0.1	5.2 ± 0.8*	1.9 ± 0.1	1.8 ± 0.1	9.1 ± 3.4
	Late Apo	1.1 ± 0.1	1.4 ± 0.5	5.8 ± 1.2*	1.1 ± 0.3	1.0 ± 0.5	26.6 ± 2.6*
	Death	2.0 ± 0.1	1.7 ± 0.3	11.8 ± 1.3*	1.1 ± 0.7	1.2 ± 0.3	30.9 ± 3.7*
48 h	Live	96.5 ± 0.4	95.5 ± 0.2	77.7 ± 1.7*	96.8 ± 0.2	96.1 ± 0.8	28.4 ± 9.8*
	Early Apo	0.9 ± 0.2	1.7 ± 0.2	4.9 ± 0.6*	1.3 ± 0.2	2.2 ± 0.8	6.9 ± 0.7*
	Late Apo	1.6 ± 0.2	1.7 ± 0.1	6.2 ± 0.2*	0.8 ± 0.3	0.6 ± 0.2	10.4 ± 1.8*
	Death	1.1 ± 0.1	1.1 ± 0.1	11.2 ± 1.3*	1.1 ± 0.1	1.1 ± 0.1	54.4 ± 8.0*
72 h	Live	94.0 ± 2.5	93.7 ± 1.4	70.4 ± 2.1*	95.9 ± 0.8	91.8 ± 0.9	24.0 ± 7.7*
	Early Apo	1.4 ± 0.2	2.1 ± 0.7	4.5 ± 0.7*	2.1 ± 0.5	3.7 ± 0.3	7.3 ± 1.3*
	Late Apo	2.1 ± 1.2	2.4 ± 0.5	12.6 ± 0.8*	1.0 ± 0.1	1.3 ± 0.2	11.1 ± 1.2*
	Death	2.6 ± 1.2	1.8 ± 0.3	12.5 ± 0.6*	1.1 ± 0.2	3.3 ± 0.4	57.6 ± 9.7*

Cells were treated with IC_50_ SCY for 24, 48, and 72 h

The results are expressed as mean ± SD of three independent experiments (*p < 0.05 compared to DMSO control, based on ordinary one-way ANOVA with Dunnett’s post hoc test)

**Fig. 7:**
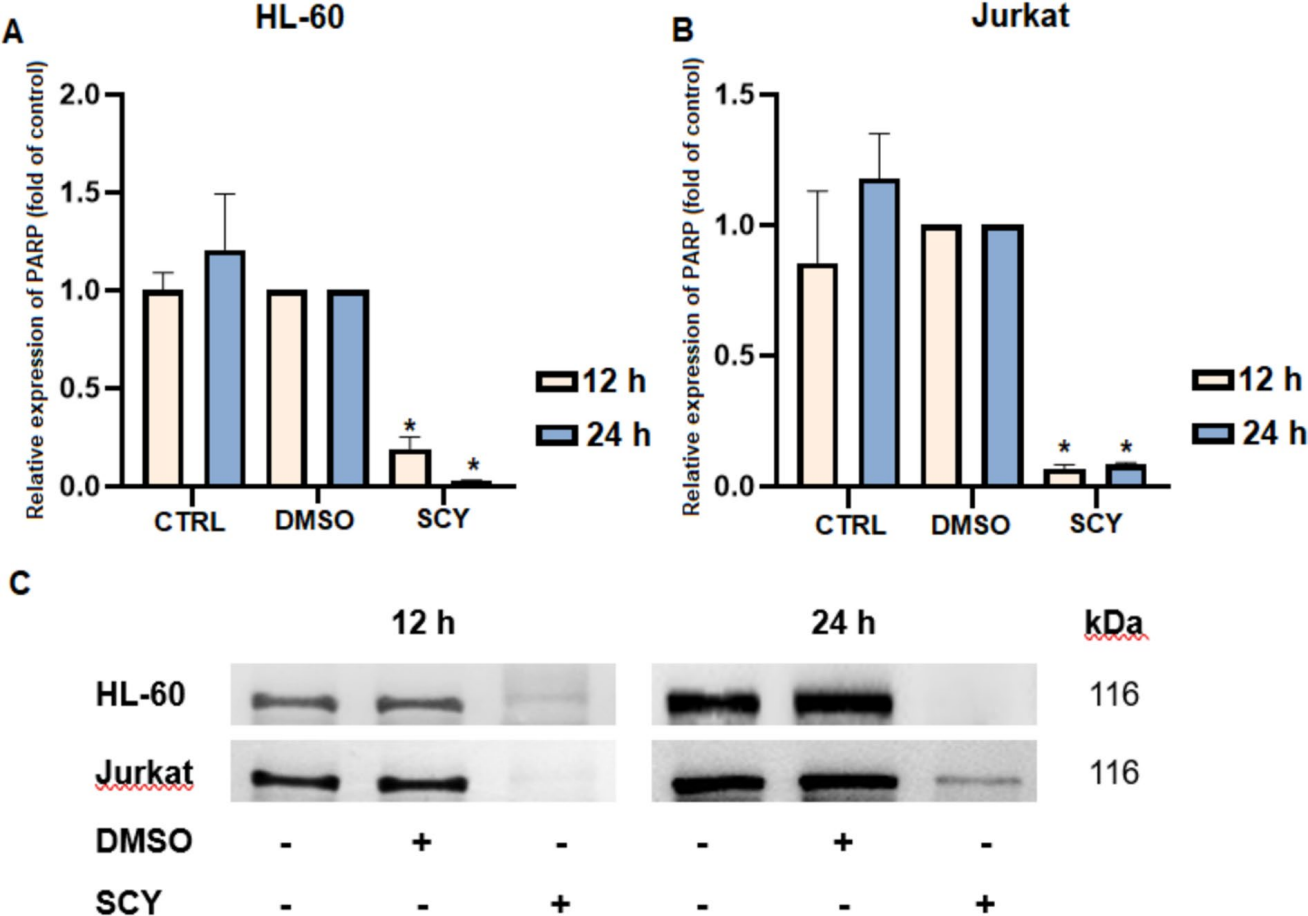
Modulation of PARP protein expression in SCY-treated leukemia cell lines. **A** Densitometry analysis of relative protein expression in HL-60 cells. **B** Densitometry analysis of relative protein expression in Jurkat cells. **C** Western blot analysis PARP expression. Results are presented as a mean ± SD of independent experiments (*p < 0.05 compared to DMSO control, based on ordinary one-way ANOVA with Dunnett’s post hoc test)

During apoptosis, the levels of the intact DNA repair protein PARP (poly-ADP-riboso-polymerase; 116 kDa) typically decrease as a result of the protein’s cleavage by executioner caspases which are activated during the apoptotic cascade. Western-blot analyses revealed a significant decrease in the levels of intact PARP protein in both cell lines 12 and 24 h after treatment with SCY (Fig. 7). Complete degradation (no visible bands) was also observed after 48 and 72 h in both cell lines (data not shown).

## Discussion

The widely distributed gram-negative photoautotrophic prokaryotes known as cyanobacteria are already seeing extensive use in the biomedical field due to the broad range of bioactive compounds which they produce. One such compound is SCY, a secondary metabolite which is present in the extracellular polysaccharide sheath of various cyanobacteria, including the genera *Nostoc*, *Scytonema*, *Lyngbya*, *Chroococcidiopsis*, and *Rivularia* [16]. As was mentioned above, scytonemin exhibits anti-proliferative, anti-inflammatory, and antioxidant activities which render the molecule a highly attractive target in the development of new medical applications.

The in vitro studies conducted in this research investigated the effect of scytonemin on cervical cancer (HeLa), colorectal cancer (HCT116 and Caco-2), breast cancer (MCF-7 and MDA-MB-231), lung cancer (A549), and acute leukemia (Jurkat and HL-60) cell lines. Resazurin assays determined that scytonemin exerted a significant inhibitory effect on all of the tested tumor lines, with the strongest effect being observed against Jurkat and HL-60 leukemia cells, with IC_50_ values of 88.2 µM and 60.5 µM, respectively. The studies also investigated the activity of *Nostoc commune* extract (NOS), but this was found to be far less effective against the tested cancer cell lines than SCY. This is possibly as a result of synergistic or antagonistic effects in the compounds comprising the extract, differences in bioavailability and cellular uptake kinetics or incompatibilities between multitarget and single target mechanisms of action, but further research is needed to confirm these hypotheses.

Flow cytometry assays confirmed that SCY exhibited an inhibitory effect on the proliferation of Jurkat and HL-60 leukemia cells. The pronounced sensitivity of Jurkat and HL-60 cell lines to scytonemin may be attributed to several leukemia-specific biological features. Firstly, these cells exhibit elevated basal levels of reactive oxygen species (ROS) and possess weakened antioxidant defenses, such as reduced glutathione and catalase activity, making them more susceptible to the oxidative stress induced by scytonemin [17, 18]. Secondly, scytonemin is a known inhibitor of MAPK signaling pathways (ERK, JNK, p38), protein chains which are commonly constitutively activated in leukemia and play critical roles in promoting cell survival and proliferation [19, 20]. As was discussed in the results section, the proliferation inhibition mediated by SCY was far more prolific against tumor cells than against healthy cells, and this is a highly attractive trait which will be of interest to research teams involved in the development of new anticancer agents. Our findings are in agreement with other studies which have reported that SCY is capable of suppressing the growth of Jurkat leukemia cells [12, 21].

Changes in cell proliferation are generally associated with cell cycle dysregulation, and our cell cycle analyses identified significant accumulations of cells in the subG0/G1 subpopulation (a characteristic marker of apoptosis) in Jurkat cell lines after 24 h of treatment with SCY, but no cell cycle arrest was observed across any of the tested time points. This latter finding appears to conflict with several other similar studies. For example, a study by Stevenson et al. showed that SCY derived from *Stigonema* sp. demonstrated antiproliferative effects against various human fibroblast and endothelial cell lines and inhibited the activity of human polo-like kinase, which plays a key role in regulating the cell cycle at the G2/M transition. Contrarily, G1 cell cycle arrest was induced in HL-60 cell lines up to 48 h of treatment with SCY [21]. Similarly, a study by Zhang et al. noted that SCY induced G2/M phase cell cycle arrest in multiple myeloma cell lines (U266, NCI-H929, and RPMI8226) [11], as did a study by Subhashini et al. (2004) into the apoptotic mechanisms induced by C-phycocyanin in chronic myeloid leukemia K562 cells [22].

Active at the G1/S cell cycle checkpoint, the Rb protein plays an important role in cell cycle regulation. When present in its hypophosphorylated active state, Rb is a tumor suppressor that inhibits cell cycle progression by binding to and repressing E2F transcription factors which are necessary for S-phase entry [23]. Our findings confirmed that SCY treatment had altered Rb protein expression and activity, leading to a decreased phosphorylation state of Rb protein in a time-dependent manner in both leukemic cell lines. This hypophosphorylation was associated with G1 cell cycle arrest in HL-60 cells treated with SCY.

Flow cytometry analyses of p21 expression showed time-dependent increases in both of the tested cell lines after SCY treatment, allowing us to conclude that Rb dephosphorylation precedes the upregulation of p21 expression. These findings suggest that Rb dephosphorylation could represent an independent or parallel mechanism of scytonemin action, one which is not directly mediated by p21 expression. The cyclin-dependent kinase inhibitor 1A (p21) prevents Rb phosphorylation by binding to the CDK2 and CDK4/6 inhibitors [24]. On this basis, we can assume that the elevated expression of p21 mediated by SCY is connected with Rb activity and G1 cell cycle arrest in HL-60 cells. Similarly, Largazole, a natural compound isolated from the marine cyanobacterium *Symploca* sp., was found to have induced cell cycle arrest in the G1 phase in A549 and NCI-H460 lung cancer cells in a process associated with p21 upregulation and Rb dephosphorylation [25]. Jiang et al. also observed p21 upregulation in MDA-MB-231 cells treated with C-phycocyanin [26].

Phosphatidylserine externalization is one of the earliest events in the programmed cell death pathway. Our flow cytometry results used Annexin V/PI staining to reveal a significant increase in the number of cells in both early and late stages of apoptosis with an increasing trend over time in both Jurkat and HL-60 cell lines. These findings tally with those of Safaei et al. who used a similar Annexin V/PI staining technique to examine the antitumor properties of C-phycocyanin isolated from the cyanobacterial strain *Limnothrix *sp*.* on the MCF-7 breast cancer cell line. Their results also showed a significant increase in cells in the early stage of apoptosis after 24 h of C-phycocyanin treatment, with the percentage of cells in the early stage of apoptosis decreasing over time and leading to late apoptosis and necrosis [27].

Changes in mitochondrial membrane potential (MMP) are also a critical factor in cell death. Decreases in MMP and outer mitochondrial membrane permeabilization release proapoptotic factors such as cytochrome *c* into the cytosol, leading to interactions with APAF-1 which form apoptosome. This activates caspase 9 and, in turn, the executioner caspases 3/7 [28, 29] which are commonly used to screen apoptosis inducers, including those derived from natural extracts [30]. Loss of MMP is a manifestation of mitochondrial membrane depolarization, and although this is a hallmark of apoptosis it cannot precede the release of cytochrome *c* release. The order of these events may depend on the cell type and stimulus [31]. Our study monitored the percentage of cells which displayed loss of mitochondrial membrane potential (MMP) and tested positive for cytochrome *c* after 24, 48 and 72 h of treatment with scytonemin. While the percentage of cells with reduced MMP gradually increased over time, the percentage of cells positive for cytochrome *c* decreased over time. There are several possible reasons for this seemingly contradictory trend; it may indicate that cytochrome *c* release occurs during earlier phases of apoptosis, or that the cells are subsequently in a phase with impaired MMP, or that cytoplasmic cytochrome *c* becomes degraded or no longer detectable over time. Since our analyses did not include assays at very early time points, it is not possible to determine fully whether the loss of MMP preceded or followed the release of cytochrome *c*.

Our results show that SCY had significantly reduced MMP and had increased cytoplasmic cytochrome *c* levels in leukemia cells, with these effects peaking at 24 h after treatment. The findings also reveal the activation of caspase 9 and caspases 3/7, along with PARP cleavage, in both Jurkat and HL-60 cells, indicating that apoptosis had occurred [32]. Similar studies with other cyanobacteria-derived substances have also reported the activation of intrinsic mitochondrial caspase-dependent apoptosis. For example, Heisnam et al. investigated the antiproliferative effect of C-phycocyanin isolated from *Westiellopsis *sp*.* cyanobacteria on the MDA-MB-231 breast cancer cell line, reporting caspase-3-dependent apoptotic cell death [33]. Similarly, a study by Permatasari et al. investigated the anticancer effects of the green seaweed *Caulerpa racemosa* on the HeLa cervical cancer cell line, with the results showing that the extract had increase the expression of cleaved caspase 3. The data obtained from Annexin V/PI analyses confirmed a reduced level of cell viability and the induction of apoptosis 24 and 48 h after the application of the substance [34]. Voráčová et al. tested the antiproliferative effects of extracts from *Nostoc *sp*.* cyanobacteria on the PaTu 8902 pancreatic cancer cell line and found that the crude extract had significantly increased the levels of caspases 3/7 in the pancreatic cancer cell line [30], a conclusion which aligns with our findings. Additionally, a study of the anticancer effects of nostatin A, a bioactive peptide isolated from *Nostoc *sp. cyanobacteria, by Delawska et al. reported that the agent had induced mitochondrial apoptosis and cell cycle arrest in the S phase, while also noting the increased activation of effector caspases 3/7 and PARP cleavage in HeLa cervical cancer cells [35].

Despite these studies, however, the potential apoptotic effect of SCY on the leukemic Jurkat and HL-60 cell lines has not been sufficiently investigated to date, with only a limited number of scientific studies addressing the apoptotic effect of scytonemin on these specific cancer cell lines. As with the other cyanobacteria-derived substances, some research suggests that certain compounds can induce apoptosis not only in hematological malignancies but also in solid tumors. Although our analyses did not directly measure reactive oxygen species (ROS) levels, studies published to date suggest that oxidative stress may contribute significantly to R-scy-induced cellular damage. For example, Itoh et al. reported that preincubation of Jurkat cells with N-acetylcysteine (NAC) reversed the inhibition of cell growth mediated by a reduced form of SCY (R-scy). Using the CellROX fluorescent probe, the authors found that treatment with R-scy led to an approximately seven-fold increase in intracellular ROS levels compared to the untreated controls, with this increase subsequently being suppressed to a significant degree by NAC [12]. These findings support the idea that an oxidative mechanism may also be involved in the cellular effects observed in our assays, although this possibility was not directly investigated in our model and will be the subject of future studies.

In general, research into the apoptotic effects of algae-derived compounds on the Jurkat and HL-60 cell lines is limited, and additional studies are needed to develop a fuller understanding of the potential of these natural substances in the treatment of leukemia. Future research should include in vivo studies using leukemia mouse models to confirm the efficacy and systemic toxicity of scytonemin under physiological conditions. Additionally, combination therapies with established chemotherapeutics should be explored to evaluate potential synergistic or sensitizing effects. Mechanistic studies focusing on key signaling pathways, such as MAPK, PI3K/AKT, and intrinsic mitochondrial apoptotic pathways, are also essential in delineating the molecular targets of scytonemin and related compounds. These future research aims will help to establish the therapeutic relevance of algae-derived compounds and support their potential integration into novel leukemia treatment strategies.

## Conclusion

The results outlined in this study have shown that SCY exerts a significant time-dependent pro-apoptotic effect on the leukemic Jurkat and HL-60 cell lines. The resazurin reduction assays determined that these two cell lines were the most sensitive to SCY, with IC_50_ values of 88.2 µM and 60.5 µM, respectively. SCY-induced cell cycle arrest in the G1 phase was observed in HL-60 cells, whereas the Jurkat cell lines showed significant accumulations of cells in the subG0/G1 sub-population, albeit without clear evidence of arrest in any specific phase. The results of the flow cytometry assays showed an increase in p21 expression and a reduction in mitochondrial membrane potential, accompanied by the release of cytochrome *c* into the cytosol, indicating activation of the intrinsic apoptotic pathway. These results also demonstrated a SCY-induced and time-dependent cleavage of the initiator caspase-9 followed by the activation of the executioner caspases 3/7. Western blot analyses confirmed a decrease in levels of intact PARP as well as a time-dependent reduction in both phosphorylated and total forms of Rb in both cell lines. These findings suggest that SCY interferes with cell cycle regulation via the p21/Rb pathway and triggers mitochondrial-mediated apoptosis. As a bioactive pigment derived from cyanobacteria, SCY is a highly promising candidate in the development of novel anticancer strategies, but further research is needed to clarify its mechanisms of action and potential use in combination therapies. Future studies should include in vivo testing of scytonemin using animal models of acute leukemia to evaluate its therapeutic potential under physiological conditions. In addition, the possibility of integrating scytonemin into conventional chemotherapeutic agents, such as cytarabine or doxorubicin, should also be explored to order to identify possible synergistic effects and enhanced efficacies. Further study into these fields could provide valuable insights into the function of this promising natural agent and optimize scytonemin-based therapies in the treatment of acute cases of leukemia.

## Data Availability

The data presented in this study can be provided by the authors on reasonable request. Plant materials are available by Dr. Kecsey.
